# From research to a political commitment to strengthen access to surgical, obstetric, and anesthesia care in Africa by 2030

**DOI:** 10.3389/fpubh.2023.1168805

**Published:** 2023-05-16

**Authors:** Pierre M'pele, Justina O. Seyi-Olajide, Tarcisse Elongo, Jorn Lemvik, Delanyo Dovlo, Emmanuel A. Ameh

**Affiliations:** Scientific Secretariat of the International Symposium on Surgical, Obstetric, and Anesthesia Systems Strengthening by 2030 in Africa, Mercy Ships Africa Office, Cotonou, Benin

**Keywords:** Africa surgical initiative, safe surgery, safe anesthesia, Dakar Declaration, Regional Action Plan

## Abstract

**Objective:**

This study aimed to engage African leaders and key stakeholders to commit themselves toward the strengthening of surgical, obstetric, and anesthesia care systems by 2030 in Africa.

**Methods:**

From research to a political commitment, a baseline assessment was performed to foster the identification of the gaps in surgical care as a first step of an inclusive process. The preliminary findings were discussed during the International Symposium on Surgical, Obstetric, and Anesthesia Systems Strengthening by 2030 in Africa. The conclusions served to draft the Dakar Declaration and its Regional Action Plan 2022–2030 to improve access to surgical care by 2030 in Africa, endorsed by Heads of State.

**Results:**

The International Symposium was composed of two meetings that gathered (i) 85 scientific experts and (ii) 28 ministers of health or representatives from 28 sub-Saharan African countries. The 28 African countries represent (i) 51% of the continent's total population, (ii) 68% of the 47 African countries of the WHO Africa Region, (iii) 58% of all African Union countries, and (vi) 79% (3,371) of the WHO Africa Region's total (4,271) health districts. The International Symposium and the Heads of State Summit successfully produced the Dakar Declaration on access to equitable, affordable, and quality Surgical, Obstetric, and Anesthesia Care by 2030 in Africa and its Regional Actions Plan 2022–2030 which prioritizes 12 urgent actions needed to be implemented, six strategic priorities, 16 key indicators, and an annual dashboard to monitor progress.

**Conclusion:**

The Dakar Declaration and its Regional Action Plan 2022–2030 are a commitment to establish quality and sustainable surgical, obstetric, and anesthesia care in each African country within the ambitious framework of “*The Africa we want*” Agenda 2063.

## Background

Over the past three decades, the African continent has made steady progress in improving public health, despite disparities between regions, countries, and within countries. Africa's exponential economic growth and development, and the additional significant contribution of global health initiatives, have facilitated this progress.

Some of the advancements in health include a 37% decline in mortality rates between 2000 and 2015 (1). Life expectancy has also grown by nearly 10 (46–56) years from 2009 to 2019 (2). However, Africa's overall performance still lags in other health indicators. These fragile gains, however, have not been matched with similar progress in health system strengthening, service integration, or hospital care, nor have they been equitably distributed among individuals of all socio-economic levels (3), mainly in the area of surgical, obstetric, and anesthesia (SOA) care.

Despite the efforts made by some Africa Union Member States with tangible achievements, the continent does not yet appear to be on track to achieve Sustainable Development Goal 3: “Health for all and promotion of wellbeing for all at all ages.”

Africa alone bears 25% of the global burden of disease and one-third of the world's clinical conditions requiring emergency care and essential surgical, obstetric, and anesthetic services (EESOACSs). Despite having 17% of the world's population, the continent has only 2% of the world's doctors and 0.7 surgical specialists per 100,000 people (4).

Every year, 16.9 million people worldwide die due to lack of access to surgical care, and 93% of sub-Saharan Africa still lacks access (5). Surgery has been a neglected component of health care for people on the African continent. Equitable integration of surgical and anesthetic care remains one of the key challenges to strengthening health systems and achieving universal health coverage in Africa.

Over the years, the focus was mainly on infectious diseases, resulting in a significant reduction in morbidity and mortality from these conditions in some African countries (6).

On the occasion of the celebration of 30 years of Mercy Ships in Africa (1990/91–2021/22), Mercy Ships seized this opportunity to consolidate and strengthen its partnership with African countries and all national and international partners involved in strengthening surgical care and to mobilize policymakers and leaders to work together to integrate and scale up surgical care in national health development strategies.

Evidence has shown that investing in and strengthening surgical care within the existing healthcare system would lead to the overall strengthening and improvement of the entire healthcare system (7, 8).

As additional efforts were needed to strengthen the provision of safe, timely, and affordable emergency and essential surgical care and anesthesia, the World Health Assembly resolution 68.15 was made (9). Mercy Ships and the government of the Republic of Senegal launched a process to engage key stakeholders to commit themselves toward strengthening surgical, obstetric, and anesthesia care systems by 2030 in Africa.

## Methods

From research to political commitment, the WHO baseline assessment, which uses two simplified tools (*country general information and district-hospital survey*), is the first step in an inclusive process. Data were collected and analyzed using the Survey Monkey^®^ platform. This was followed by a strategic analysis and guidance involving African experts and Ministers of Health gathered in an International Symposium. Six Heads of state then endorsed the Dakar Declaration and its Regional Action Plan to improve access to surgical care in Africa by 2030.

**Figure 1:**
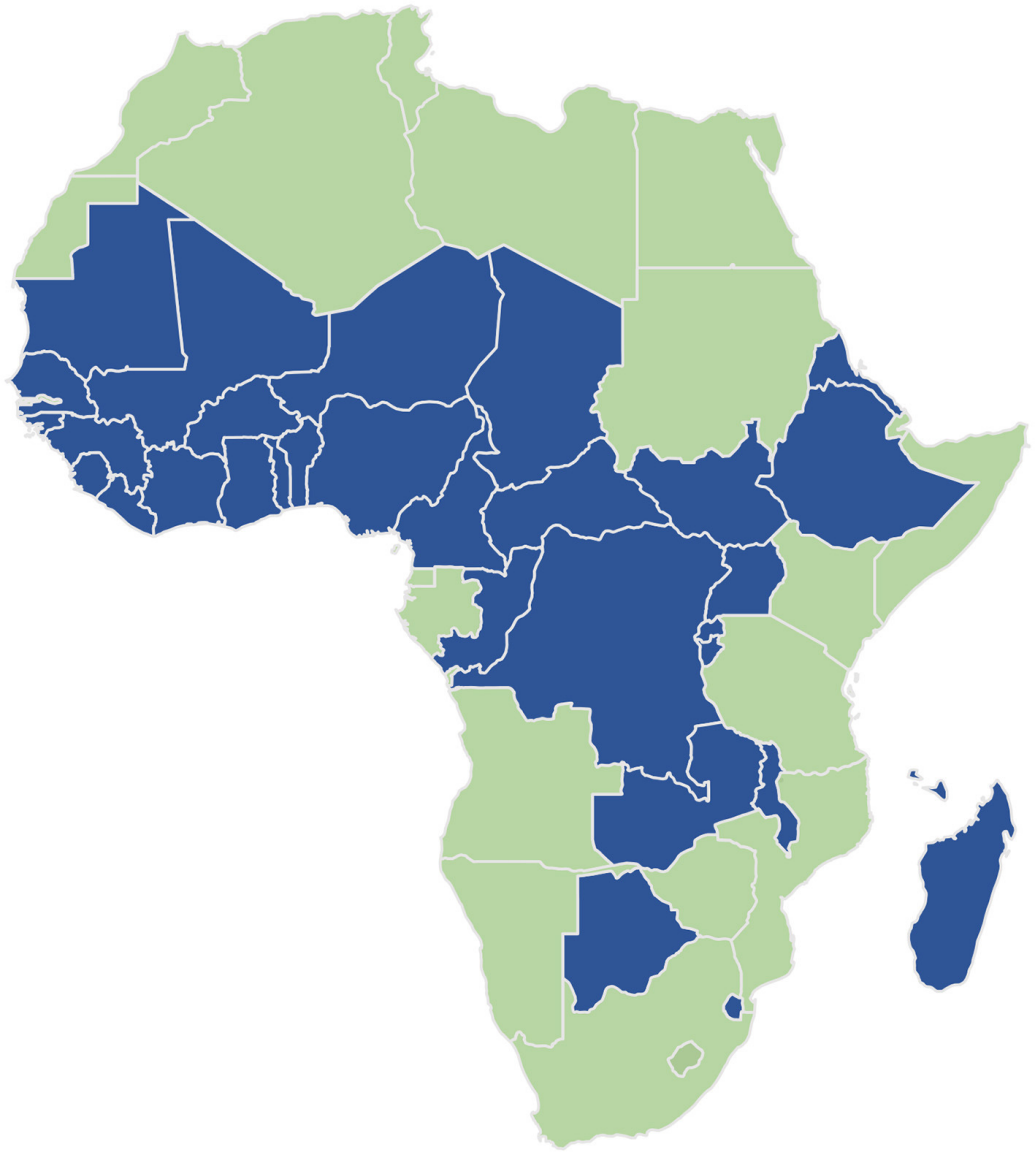
Thirty-two sub-Saharan African countries involved in the baseline assessment.

(i) The baseline assessment aimed to foster the identification of the gaps in surgical care. The assessment was conducted in 6 months (January–July 2022) in 601 district hospitals (Table 1) in 32 sub-Saharan African countries (Figure 1) and performed by more than 600 national investigators and coordinators from the Ministry of Health at the national level and investigators at the district-hospital level. As in similar studies or surveys, refereeing the AFRO guidelines, the sample size was set at 20% of the total number of health districts for each selected country (10). The health districts were selected from all the regions of the country to ensure representative geographical coverage with a convenient sample of 20% of district hospitals in each country. In three countries (D R Congo, Madagascar, and Botswana), the district hospitals surveyed did not reach 20% because of security issues and flooding as well as incomplete data.

**Table 1:** Baseline assessment in 601 district hospitals.

**Serial no**.	**Countries**	**No. of health districts**	**20% of district hospitals assessed**
1.	Benin	77	16
2.	Burkina Faso	70	14
3.	Burundi	47	9
4.	Botswana	24	3
5.	Cameroun	190	38
6.	CAR	35	7
7.	Congo	52	10
8.	Comoros	17	3
9.	Côte d'Ivoire	113	22
10.	DR Congo	480	32^*^
11.	Eritrea	58	12
12.	Eswatini	4	1
13.	Ethiopia	123	25
14.	Gambia	7	1
15.	Ghana	260	50
16.	Guinea	38	8
17.	Guinea-Bissau	11	3
18.	Liberia	15	5
19.	Madagascar	114	19^*^
20.	Malawi	28	6
21.	Mali	75	15
22.	Mauritania	57	7
23.	Niger	72	14
24.	Nigeria	774	157
25.	Rwanda	30	7
26.	Senegal	79	15
27.	Sierra Leone	16	3
28.	South Sudan	80	16
29.	Chad	129	26
30.	Togo	44	9
31.	Uganda	136	26
32.	Zambia	116	22
**Total**		**3,371**	**601**

^*^In these countries, the number of district hospitals surveyed did not reach 20%.

**Figure 2:**
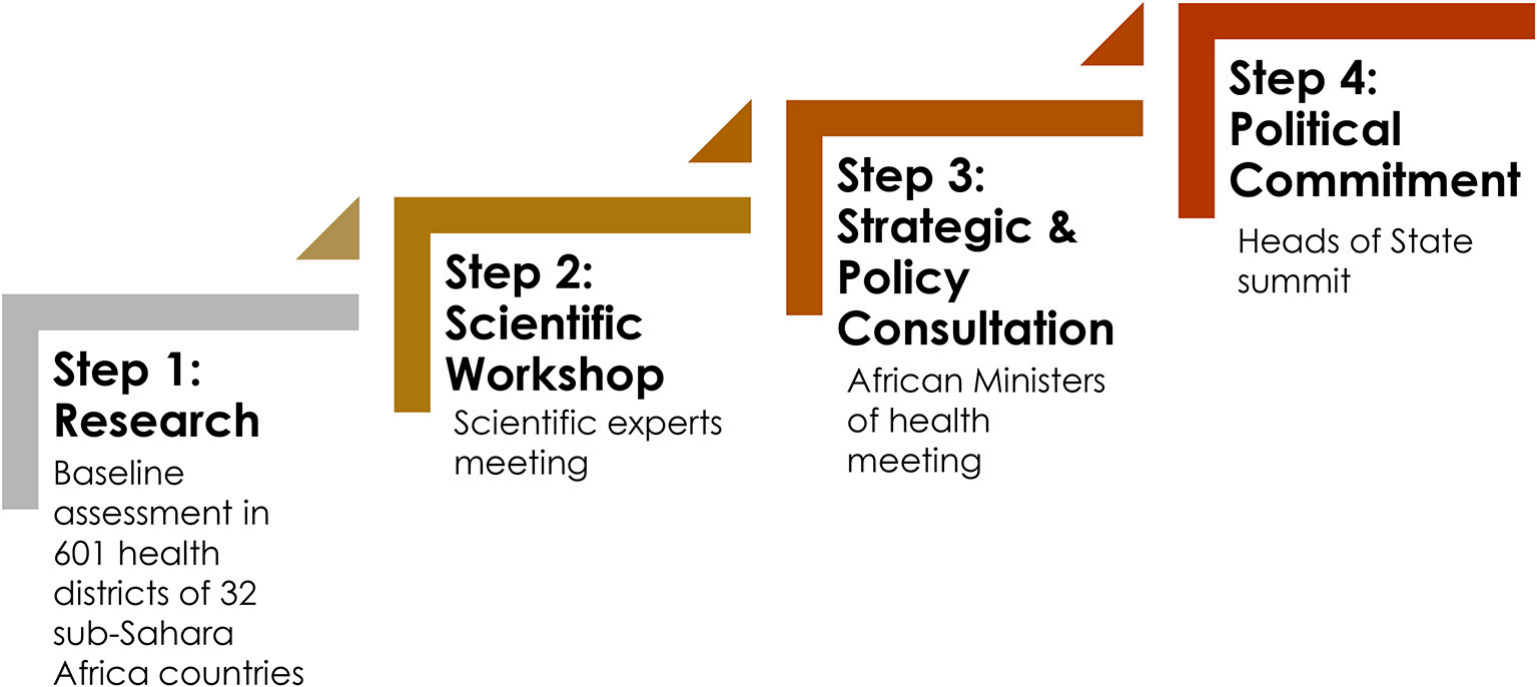
Dakar Declaration: steps from research to political commitment.

Data were collected in the following areas: infrastructure, human resources, service delivery, information management, finance, impact of COVID-19 on surgery, governance, and leadership and children's surgery and uploaded into the survey monkey platform. The survey was pretested in two countries before final deployment. (ii) The International Symposium (IS) was organized by the Government of Senegal and Mercy Ships in close collaboration with the WHO Regional Office for Africa and in partnership with various international and regional organizations and African key actors of the health sector at all levels. The IS gathered participants from 28 African countries, namely, Benin, Burundi, Burkina Faso, Cameroon, Central African Republic, Chad, Congo, Comoros, Côte d'Ivoire, Ethiopia, Eswatini, Gambia, Ghana, Guinea, Guinea-Bissau, Liberia, Madagascar, Mali, Malawi, Mauritania, Niger, Nigeria, Senegal, Seychelles, Sierra Leone, South Sudan, Uganda, and Togo (Figure 1), and Somaliland as an observer and was held in two phases: (a) The Experts Meeting identified and agreed on the key findings of the baseline assessment, formulated priority recommendations, proposed a roadmap 2022–2030 for scaling up and investing in the strengthening of surgical, obstetric, anesthesia, and nursing care in Africa, and developed a draft Declaration of commitment that was submitted to the Ministers of Health meeting. (b) The African Ministers of Health discussed and adopted a Declaration of commitment to access to equitable, affordable, and quality surgical, obstetric, and anesthesia care in Africa, as well as a draft Regional Action Plan 2022–2030, a roadmap for the scale-up and investment in the strengthening of surgical, obstetric, anesthesia, and nursing care in Africa by 2030, including a monitoring and evaluation plan. (iii) On 30 May 2022 in Dakar, Senegal, the Heads of State Summit of six countries (Cameroon, Congo, Comoros, Gambia, Guinea-Bissau, and Senegal) endorsed the Dakar Declaration on Access to Equitable, Affordable, and Quality Surgical, Obstetric, and Anesthesia Care in Africa by 2030, commonly referred to as “The Dakar Declaration,” and its Regional Action Plan 2022–2030 (Figure 2). President of Senegal, Macky Sall, and current Chairperson of the African Union will submit the Dakar Declaration to the African Union Heads of States ordinary summit in February 2023.

## Results

The preliminary findings of the baseline assessment show that the surgical care and national health systems in the majority of the countries assessed are disorganized, weak, and fragile. Few countries have created “National Surgical, Obstetric, Anesthesia and Nursing Plans (NSOAPs)” to guide and strengthen surgical care, and most of Africa remains underserved in terms of surgical care. The baseline assessment identified the ongoing challenges to universal health coverage from deficiencies in surgical, obstetric, anesthetic, and related care due to (1) workforce deficits in the core human resources needed for surgical services, (2) significant infrastructure and equipment deficits and disparity within countries, (3) lack of service delivery due to weaknesses in the core and support services required to deliver safe, surgical care, (4) challenges of financing surgical, obstetric, and anesthesia services as part of national health strategies, (5) lack of regulation and governance structures for surgical care at all levels, (6) information, (7) inadequate health promotion and prevention efforts on the causes of morbidity and mortality from surgical and related conditions, and (8) lack of leadership and management of surgical, obstetric, and anesthesia care. The Dakar Declaration contains nine key commitments (Table 2). The Regional Action Plan 2022–2030, the result of the political commitment, is an 8-year workplan for all African countries to upgrade their surgical care system by 2030. The Regional Action Plan prioritizes 12 urgent actions (Table 3), six strategic priorities, 16 key indicators, and an annual dashboard to monitor progress (Table 4).

**Table 2:** Nine key commitments of the Dakar Declaration.

	**Dakar Declaration commitments**
1	**WE CALL UPON** all Nations of the Africa Region, key national and international partners, and stakeholders in surgical safety to commit the implementation of the 12 urgent actions needed as per the annex 1 of this declaration.
2	**WE COMMIT** to involve Governments, Legislatures, Ministries of Health, Ministries of Finance, and supported by key stakeholders including educators, trainers, and mentors of surgical, obstetric and anesthesia workforce, professional health associations and societies, academic institutions, local and international partners, health professions regulating bodies, civil society, and patient advocacy groups.
3	**WE COMMIT OURSELVES** for the scaling up and investment in the Strengthening of Surgical, Obstetric and Anesthesia Care in Africa toward the implementation of the Regional Action Plan 2022–2030.
4	**WE RECOGNIZE** that surgery has been a neglected component of national health systems and that Nations share common challenges including infrastructure, human resources, financing, and strategic vision.
5	**WE COMMIT OURSELVES** toward meeting the target of the Abuja Declaration in allocating 15% of a national budget to health.
6	**WE COMMIT OURSELVES** to advocate for the creation of a regional fund (similar to the Global Fund for AIDS, Malaria and Tuberculosis) to boost resource mobilization to strengthen access to Surgical, Obstetric and Anesthesia Care.
7	**WE COMMIT** to implement the 2022–2030 roadmap with its strategic priorities, key indicators and dashboard as per the annex 2 of this declaration.
8	**WE ENDORSE** the conclusions of the 28 African Ministers of Health of the WHO Africa Region meeting in Dakar, Republic of Senegal, on May 6th, 2022 and the setting-up of an African Scientific Working Group to oversight the implementation of the Regional Action Plan.
9	**WE RESOLVE** to gather every 2 years between now and 2030 to assess progress, to exchange ideas and innovations, and to share experience between and among countries.

**Table 3:** Twelve (12) urgent actions needed to be implemented by 2030.

**N°**	**Twelve urgent actions needed**
1	Urgently expand core and support services workforce needed to provide safe surgical care and expand pre- and post-service trainings and professional development programs;
2	Urgently increase health infrastructure and equipment that enhances access to good quality and safe surgical care for our population especially the most vulnerable and deprived communities;
3	Improve the financial investment into expanded surgical services and that improves access and reduces financial barriers, and removes risk of financial impoverishment for vulnerable groups including children, women and the disabled;
4	Establish structures to improve governance, leadership, and management of surgical, obstetric, anesthetic, and related services as part of Universal Health Coverage;
5	Engage with communities to prevent the causes of morbidity and mortality related to surgical and related conditions and the promotion in our populations of healthy lifestyles;
6	Streamline actions to relieve our populations of the high disease burden posed by surgical, obstetric, and anesthetic deficiency;
7	Mobilize resources from domestic and external sources to expand necessary investments into surgical services and achieve financial risk protection from accessing surgical, obstetric and anesthesia services;
8	Build workforce capacity through training programs and mentoring, to increase essential surgical procedures and interventions in each country by 2030;
9	Improve health information systems to facilitate the use of surgical data for innovation and improvement of surgical services;
10	Create and expand regional, national, and international partnerships for both technical and resource mobilization;
11	Incorporate gender equity into National Surgical, Obstetric, Anesthesia Plans with clear indicators;
12	Integrate essential surgical, obstetric and anesthesia interventions, indicators, and budgets into national health sector policies, strategies, and plans.

**Table 4:** Regional action plan strategic priorities, key indicators, and annual dashboard.

**N°**	**Strategic priorities (6)**	**Key indicators (16)**	**Annual dashboard**
1	Governance and leadership	1.1 National Health Strategy, National Surgical, • Obstetrics and Anesthesia Plan; 1.2 100% of countries should have launched NSOAP and commenced implementation; 1.3 Annual National Surgical meeting for countries to report and track progress; 1.4 Setting up of Africa Scientific Task Force for capacity building, support to implementation and monitoring and evaluation of progress.	National Surgical, Obstetric and Anesthesia Plan, Annual National Surgical Meeting
2	Human ressources	At least 50% should have surgical/obstetric and anesthesia provider 24/7.	24/7 availability of surgical, obstetric and anesthesia provider
3	Infrastructure	3.1 100% should have functional operating rooms; 3.2 100% of should have oxygen available 24/7; 3.3 100% availability of pulse oximetry in operating rooms.	(i) Functional operating rooms, permanent access to oxygen, pulse oximetry availability
4	Service delivery	4.1 At least 50% 2-h access to facility with surgical care; 4.2 100% 2 h access to safe blood supply; 4.3 100% use of safe surgery checklist; 4.4 At least 50% of hospitals should have IPC programmes: 4.5 100% tracking of perioperative mortality.	Access to care, blood delivery, use of the WHO surgical safety checklist, peri-operative mortality rate, infection control and prevention programme
5	Health information and research	5.1 100% should have reliable and durable health record system; 5.2 50% should have electronic health record system	Emergency and essential surgical care, pediatric surgeons,
6	Finance	6.1 50% financial risk protection for surgical care; 6.2 Funding provided for research in 100% of hospitals.	Financial risk protection

## Discussion

The current focus on health systems and service delivery is not aligned with an effective policy response in Africa (7, 11). Many priorities are still partner-driven, with limited policy or institutional buy-in. The verticalization of the efforts predominates, both for health services and health system strengthening initiatives, with limited linkages within and across the different areas. The main consequence being a fragmented service delivery without guarantee of the availability of essential services.

There is also a weak emphasis on an integrated approach to the system-strengthening efforts, which leads to duplications and, in some cases, underinvestment in critical elements needed for effective service provision.

In this context, effective leadership has paramount importance to manage new change initiatives and implement national reforms within the health sectors. Leaders have a key role to facilitate mechanisms for making policies, managing the sector, and producing as well as accounting for results from health interventions in the six building blocks of the health systems as defined by the WHO. In those conditions, the health governance area represents a scope of actions across all domains providing policies, standards, regulations, and guidance to control the use of resources and the functioning of health systems (12).

Taking forward the implementation of multiple commitments made at regional and international levels is challenging in Africa, as countries are complex and exhibit wide variations in their system focus, design, and performance. For example, the Abuja Declaration made in 2001 which aims to dedicate 15% of the national budget to the health sector is implemented only by a few African countries (13, 14). The main consequence being a fragmented service delivery without a guarantee of the availability of essential services. Therefore, the Abuja Declaration is still necessary for the successful implementation of the Dakar Declaration to upgrade and scale up all needed interventions to build strong and resilient national health systems.

The Dakar Declaration is a joint initiative bringing together the government of Senegal and Mercy Ships with the participation of key regional and international partners involved in the surgical, obstetric, and anesthesia care system strengthening in Africa.

## Conclusion

To solve the current surgical care crisis, African leaders must commit to significant and focused efforts to improve the availability, quality, and affordability of surgery in the continent and then within their countries.

These efforts should be supervised by heads of state and implemented by the ministries of health in close collaboration with relevant stakeholders, including, but not limited to, the private sector, the non-governmental sector, professional organizations, and not-for-profit sectors. The culmination of the efforts needed has led to a Regional Action Plan (RAP) 2022–2030 and then to national surgical, obstetric, and anesthesia plan (NSOAP) as part of the National Health Plan (NHP). Both the Regional Action Plan and the NSOAPs will guide the integration at regional and national levels into the Africa Health Strategy 2016–2030 and into the national health policy, strategy, or plan, as there is now global and regional recognition that health systems planning is incomplete if the surgical system is left unaddressed. The Dakar Declaration and its Regional Action Plan, therefore, are a commitment as a clear pathway to building quality and sustainable surgical, obstetric, and anesthesia systems in Africa and in each country within the ambitious framework of “*The Africa we want*” Agenda 2063 (13). In the era of the Declaration of Astana on PHC for UHC and SDGs (October 2018), the surgical service should be an integral part of its first component, which is “integrated packages of quality services and considering essential public health functions” (15). The definition of essential service packages should also include surgical services so that the health system can respond easily to the needs of the communities. As a reminder, “The Africa we want” is a shared framework for inclusive growth and sustainable development for Africa to be realized in the next 50 years. It is a continuation of the Pan-African drive over centuries, for unity, self-determination, freedom, progress, and collective prosperity pursued under the African Renaissance.

## Author contributions

PM'p, JS-O, TE, JL, DD, and EA contributed to the conception, writing, and final approval of the manuscript for publication. All authors contributed to the article and approved the submitted version.
